# The Care of Colds

**Published:** 1901-06

**Authors:** 


### Abstracts and Selections.

---

### The Care of Colds.

"The Physiologic Care of Colds" is the subject of an interesting and instructive article in the April 27th number of the *Journal of the American Medical Association,* by Dr. Charles H. Shepherd. Dr. Shepherd takes a common sense view of the etiology and treatment of "colds." He thinks the part played by exposure and the like in their causation is unimportant, and that the real causative factors are such predisposing ones as overeating, insufficient exercise, want of wholesome food, and the breathing of foul air. In other words, some violation of the laws of health.

He thinks the condition called a "cold" is always one of repletion, and that, therefore, depletive measures are indicated in its treatment, hence the proverb "stuff a cold and starve a fever" is an absurdity. He advises a limited and carefully regulated diet, the drinking of large quantities of water, the administration of laxatives, and the use of the Turkish bath, followed if convenient by an oil rub. He objects strongly to the uses of quinine on the ground that it debilitates the nervous system and depresses the heart's action, and to the use of alcohol in any form because of its tendency to primarily irritate and later depress the nervous system.

